# *O*,*O*′-Di-*p*-tolyl­pyrophospho­ric bis­(dimethyl­amide)

**DOI:** 10.1107/S1600536810002692

**Published:** 2010-01-27

**Authors:** Mehrdad Pourayoubi, Saied Ghadimi, Ali Asghar Ebrahimi Valmoozi

**Affiliations:** a Department of Chemistry, Ferdowsi University of Mashhad, Mashhad, 91779, Iran; b Department of Chemistry, Imam Hossein University, P.O. Box 16575-347, Tehran, Iran

## Abstract

The title compound, C_18_H_26_N_2_O_5_P_2_, was obtained accidently from the reaction between *N*,*N*-dimethyl­phospho­ramido­chloridic acid 4-methyl phenyl ester, NaNO_2_ and 18-crown-6 in acetonitrile under reflux conditions. The asymmetric unit contains one half-mol­ecule, the complete mol­ecule being generated by crystallographic twofold symmetry, with the bridging O atom lying on the rotation axis. The P atoms exhibit a tetra­hedral coordination and are bridged *via* one O atom [P—O—P angle = 130.00 (19)°].

## Related literature

For related structures, see: Ghadimi *et al.* (2007 ▶, 2009 ▶); Pourayoubi *et al.* (2007 ▶). 
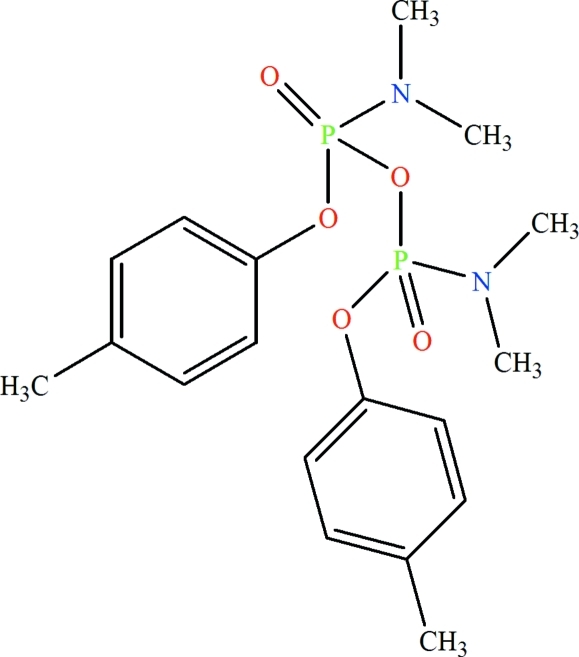


## Experimental

### 

#### Crystal data

C_18_H_26_N_2_O_5_P_2_
*M* *_r_* = 412.35
Monoclinic, 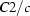

*a* = 26.484 (5) Å
*b* = 7.4195 (15) Å
*c* = 11.096 (2) Å
β = 112.949 (4)°
*V* = 2007.8 (7) Å^3^
*Z* = 4
Mo *K*α radiation
μ = 0.25 mm^−1^
*T* = 100 K
0.50 × 0.25 × 0.10 mm

#### Data collection

Bruker APEXII CCD area-detector diffractometer
Absorption correction: multi-scan (*SADABS*; Bruker, 2005 ▶) *T*_min_ = 0.930, *T*_max_ = 0.978
6483 measured reflections
2415 independent reflections
1763 reflections with *I* > 2σ(*I*)
*R*_int_ = 0.039

#### Refinement

*R*[*F* ^2^ > 2σ(*F* ^2^)] = 0.052
*wR*(*F* ^2^) = 0.124
*S* = 0.94
2415 reflections
126 parameters
H-atom parameters constrained
Δρ_max_ = 0.34 e Å^−3^
Δρ_min_ = −0.37 e Å^−3^

### 

Data collection: *APEX2* (Bruker, 2005 ▶); cell refinement: *SAINT* (Bruker, 2005 ▶); data reduction: *SAINT*; program(s) used to solve structure: *SHELXTL* (Sheldrick, 2008 ▶); program(s) used to refine structure: *SHELXTL*; molecular graphics: *SHELXTL*; software used to prepare material for publication: *SHELXTL*.

## Supplementary Material

Crystal structure: contains datablocks I, global. DOI: 10.1107/S1600536810002692/bg2328sup1.cif

Structure factors: contains datablocks I. DOI: 10.1107/S1600536810002692/bg2328Isup2.hkl

Additional supplementary materials:  crystallographic information; 3D view; checkCIF report

## Figures and Tables

**Fig. 1.**
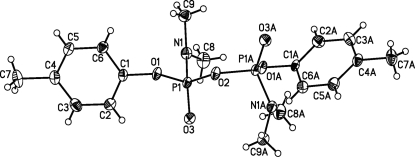
Molecular view (50 % probability level) of the title compound. Symmetry code A: -x,y,-z+1/2.

## supplementary crystallographic information

### Comment

Following our previous works about amido phosphoric acid esters with general formula [(CH_3_)_2_N][*p*-CH_3_—C_6_H_4_—O]P(O)X [for example X = NHCH(CH_3_)_2_ (Pourayoubi *et al.*, 2007) and NHC(CH_3_)_3_ (Ghadimi *et al.*, 2009)], we report here on the synthesis and crystal structure of title compound, [(CH_3_)_2_N][*p*-CH_3_—C_6_H_4_—O]P(O)(O)P(O)[O—C_6_H_4_-*p*-CH_3_][N(CH_3_)_2_]. The asymmetric unit contains one half-molecule, the complete molecule (Fig. 1) being generated by a twofold rotation axis. The phosphorous atoms exhibit a tetrahedral coordination and are bridged *via* one O atom (P—O—P angle = 130.0 (2)°). The bond angles around the P atoms are in the range of 94.25 (12)° (for O1—P1—O2 angle) to 117.71 (12)° (for O3—P1—O1 angle). The nitrogen atom indicates some deviation from planarity, the sum of the surrounding angles around N atom being about 353.3°.

### Experimental

[(CH_3_)_2_N]P(O)Cl[O—C_6_H_4_-*p*-CH_3_] was synthesized according to the literature method (Ghadimi *et al.*, 2007). The title compound was prepared according to the following procedure: A mixture of [(CH_3_)_2_N]P(O)Cl[O—C_6_H_4_-*p*-CH_3_] (0.82 g, 3.5 mmol), NaNO_2_ (0.24 g, 3.5 mmol) and 18-crown-6 (0.20 g) in acetonitrile (30 ml) was refluxed for 4 h and then filtered. The solvent was removed under vacuum and the solid recrystallized in a mixture of chloroform and n-hexane to produce single crystals after a slow evaporation at room temperature. IR (KBr, cm^-1^): 2995, 2900, 2880, 2820, 1850, 1580, 1480, 1440, 1300, 1235, 1250, 1185, 1100, 990, 940, 730.

### Refinement

The H(C) atom positions were calculated. All hydrogen atoms were refined in isotropic approximation in riding model with the Uiso(H) parameters equal to 1.2 Ueq(Ci), for methyl groups equal to 1.5 Ueq(Cii), where U(Ci) and U(Cii) are respectively the equivalent thermal parameters of the carbon atoms to which corresponding H atoms are bonded.

### Crystal data

**Table:**

C_18_H_26_N_2_O_5_P_2_	*F*(000) = 872
*M**_r_* = 412.35	*D*_x_ = 1.364 Mg m^−^^3^
Monoclinic, *C*2/*c*	Mo *K*α radiation, λ = 0.71073 Å
Hall symbol: -C 2yc	Cell parameters from 1898 reflections
*a* = 26.484 (5) Å	θ = 2.9–30.7°
*b* = 7.4195 (15) Å	µ = 0.25 mm^−^^1^
*c* = 11.096 (2) Å	*T* = 100 K
β = 112.949 (4)°	Plate, colorless
*V* = 2007.8 (7) Å^3^	0.50 × 0.25 × 0.10 mm
*Z* = 4	

### Data collection

**Table:**

Bruker APEXII CCD area-detector diffractometer	2415 independent reflections
Radiation source: fine-focus sealed tube	1763 reflections with *I* > 2σ(*I*)
graphite	*R*_int_ = 0.039
phi and ω scans	θ_max_ = 28.0°, θ_min_ = 1.7°
Absorption correction: multi-scan (*SADABS*; Bruker, 2005)	*h* = −34→21
*T*_min_ = 0.930, *T*_max_ = 0.978	*k* = −9→9
6483 measured reflections	*l* = −14→14

### Refinement

**Table:**

Refinement on *F*^2^	Primary atom site location: structure-invariant direct methods
Least-squares matrix: full	Secondary atom site location: difference Fourier map
*R*[*F*^2^ > 2σ(*F*^2^)] = 0.052	Hydrogen site location: inferred from neighbouring sites
*wR*(*F*^2^) = 0.124	H-atom parameters constrained
*S* = 0.94	*w* = 1/[σ^2^(*F*_o_^2^) + (0.013*P*)^2^ + 16.2989*P*] where *P* = (*F*_o_^2^ + 2*F*_c_^2^)/3
2415 reflections	(Δ/σ)_max_ = 0.002
126 parameters	Δρ_max_ = 0.34 e Å^−^^3^
0 restraints	Δρ_min_ = −0.37 e Å^−^^3^

### Special details

**Table:**

Geometry. All esds (except the esd in the dihedral angle between two l.s. planes) are estimated using the full covariance matrix. The cell esds are taken into account individually in the estimation of esds in distances, angles and torsion angles; correlations between esds in cell parameters are only used when they are defined by crystal symmetry. An approximate (isotropic) treatment of cell esds is used for estimating esds involving l.s. planes.
Refinement. Refinement of *F*^2^ against ALL reflections. The weighted *R*-factor wR and goodness of fit *S* are based on *F*^2^, conventional *R*-factors *R* are based on *F*, with *F* set to zero for negative *F*^2^. The threshold expression of *F*^2^ > σ(*F*^2^) is used only for calculating *R*-factors(gt) etc. and is not relevant to the choice of reflections for refinement. *R*-factors based on *F*^2^ are statistically about twice as large as those based on *F*, and *R*- factors based on ALL data will be even larger.

### Fractional atomic coordinates and isotropic or equivalent isotropic displacement parameters (Å^2^)

**Table:**

	*x*	*y*	*z*	*U*_iso_*/*U*_eq_	
P1	0.05951 (3)	0.15216 (10)	0.29554 (7)	0.01597 (17)	
O1	0.08997 (8)	−0.0313 (3)	0.2957 (2)	0.0188 (4)	
O2	0.0000	0.0607 (4)	0.2500	0.0198 (6)	
O3	0.07504 (8)	0.2488 (3)	0.4196 (2)	0.0224 (5)	
N1	0.06400 (10)	0.2733 (3)	0.1781 (2)	0.0192 (5)	
C1	0.14675 (11)	−0.0402 (4)	0.3240 (3)	0.0159 (6)	
C2	0.18495 (13)	0.0402 (4)	0.4337 (3)	0.0237 (7)	
H2A	0.1735	0.1112	0.4898	0.028*	
C3	0.24040 (13)	0.0160 (4)	0.4612 (3)	0.0250 (7)	
H3A	0.2669	0.0709	0.5369	0.030*	
C4	0.25790 (12)	−0.0867 (4)	0.3802 (3)	0.0205 (6)	
C5	0.21822 (12)	−0.1656 (4)	0.2707 (3)	0.0222 (6)	
H5A	0.2294	−0.2372	0.2145	0.027*	
C6	0.16261 (12)	−0.1427 (4)	0.2411 (3)	0.0205 (6)	
H6A	0.1360	−0.1965	0.1652	0.025*	
C7	0.31835 (12)	−0.1118 (5)	0.4103 (4)	0.0305 (8)	
H7A	0.3238	−0.1263	0.3284	0.046*	
H7B	0.3317	−0.2194	0.4647	0.046*	
H7C	0.3387	−0.0059	0.4574	0.046*	
C8	0.05658 (14)	0.4694 (4)	0.1789 (4)	0.0288 (7)	
H8A	0.0723	0.5265	0.1217	0.043*	
H8B	0.0751	0.5151	0.2683	0.043*	
H8C	0.0174	0.4974	0.1472	0.043*	
C9	0.04811 (13)	0.1948 (4)	0.0462 (3)	0.0239 (7)	
H9A	0.0670	0.2590	−0.0013	0.036*	
H9B	0.0084	0.2062	−0.0016	0.036*	
H9C	0.0584	0.0672	0.0538	0.036*	

### Atomic displacement parameters (Å^2^)

**Table:**

	*U*^11^	*U*^22^	*U*^33^	*U*^12^	*U*^13^	*U*^23^
P1	0.0134 (3)	0.0175 (3)	0.0189 (4)	−0.0003 (3)	0.0084 (3)	−0.0005 (3)
O1	0.0150 (10)	0.0186 (10)	0.0268 (11)	−0.0006 (8)	0.0127 (9)	−0.0007 (9)
O2	0.0159 (14)	0.0197 (15)	0.0274 (16)	0.000	0.0123 (13)	0.000
O3	0.0209 (11)	0.0254 (11)	0.0212 (11)	0.0015 (9)	0.0087 (9)	−0.0031 (9)
N1	0.0183 (12)	0.0189 (12)	0.0216 (13)	−0.0024 (10)	0.0090 (11)	0.0008 (10)
C1	0.0142 (13)	0.0140 (12)	0.0219 (15)	−0.0007 (11)	0.0097 (11)	0.0043 (11)
C2	0.0247 (16)	0.0254 (15)	0.0223 (16)	0.0021 (13)	0.0104 (13)	−0.0029 (13)
C3	0.0200 (15)	0.0249 (15)	0.0247 (17)	−0.0016 (13)	0.0029 (13)	−0.0023 (13)
C4	0.0177 (14)	0.0172 (13)	0.0276 (16)	0.0010 (11)	0.0098 (13)	0.0077 (12)
C5	0.0208 (15)	0.0224 (15)	0.0266 (16)	0.0028 (12)	0.0127 (13)	−0.0007 (13)
C6	0.0206 (14)	0.0200 (14)	0.0221 (15)	−0.0004 (12)	0.0096 (12)	−0.0033 (12)
C7	0.0168 (15)	0.0286 (17)	0.042 (2)	0.0034 (13)	0.0075 (14)	0.0097 (15)
C8	0.0291 (17)	0.0205 (15)	0.0381 (19)	−0.0004 (13)	0.0147 (15)	0.0045 (14)
C9	0.0241 (15)	0.0279 (16)	0.0221 (16)	−0.0029 (13)	0.0115 (13)	0.0008 (13)

### Geometric parameters (Å, °)

**Table:**

P1—O3	1.462 (2)	C4—C5	1.388 (4)
P1—O1	1.582 (2)	C4—C7	1.514 (4)
P1—O2	1.6059 (14)	C5—C6	1.389 (4)
P1—N1	1.625 (3)	C5—H5A	0.9500
O1—C1	1.413 (3)	C6—H6A	0.9500
O2—P1^i^	1.6059 (14)	C7—H7A	0.9800
N1—C8	1.468 (4)	C7—H7B	0.9800
N1—C9	1.476 (4)	C7—H7C	0.9800
C1—C6	1.379 (4)	C8—H8A	0.9800
C1—C2	1.379 (4)	C8—H8B	0.9800
C2—C3	1.390 (4)	C8—H8C	0.9800
C2—H2A	0.9500	C9—H9A	0.9800
C3—C4	1.389 (4)	C9—H9B	0.9800
C3—H3A	0.9500	C9—H9C	0.9800
			
O3—P1—O1	117.71 (12)	C4—C5—H5A	119.1
O3—P1—O2	112.64 (10)	C6—C5—H5A	119.1
O1—P1—O2	94.25 (12)	C1—C6—C5	118.7 (3)
O3—P1—N1	113.71 (13)	C1—C6—H6A	120.6
O1—P1—N1	106.27 (12)	C5—C6—H6A	120.6
O2—P1—N1	110.55 (11)	C4—C7—H7A	109.5
C1—O1—P1	122.57 (17)	C4—C7—H7B	109.5
P1—O2—P1^i^	130.00 (19)	H7A—C7—H7B	109.5
C8—N1—C9	114.2 (3)	C4—C7—H7C	109.5
C8—N1—P1	119.4 (2)	H7A—C7—H7C	109.5
C9—N1—P1	119.7 (2)	H7B—C7—H7C	109.5
C6—C1—C2	121.2 (3)	N1—C8—H8A	109.5
C6—C1—O1	116.9 (3)	N1—C8—H8B	109.5
C2—C1—O1	121.8 (3)	H8A—C8—H8B	109.5
C1—C2—C3	119.1 (3)	N1—C8—H8C	109.5
C1—C2—H2A	120.5	H8A—C8—H8C	109.5
C3—C2—H2A	120.5	H8B—C8—H8C	109.5
C4—C3—C2	121.3 (3)	N1—C9—H9A	109.5
C4—C3—H3A	119.4	N1—C9—H9B	109.5
C2—C3—H3A	119.4	H9A—C9—H9B	109.5
C5—C4—C3	117.9 (3)	N1—C9—H9C	109.5
C5—C4—C7	121.0 (3)	H9A—C9—H9C	109.5
C3—C4—C7	121.0 (3)	H9B—C9—H9C	109.5
C4—C5—C6	121.8 (3)		
			
O3—P1—O1—C1	−64.5 (2)	P1—O1—C1—C6	−133.6 (2)
O2—P1—O1—C1	177.1 (2)	P1—O1—C1—C2	50.4 (3)
N1—P1—O1—C1	64.3 (2)	C6—C1—C2—C3	−0.4 (5)
O3—P1—O2—P1^i^	66.93 (11)	O1—C1—C2—C3	175.5 (3)
O1—P1—O2—P1^i^	−170.64 (9)	C1—C2—C3—C4	0.2 (5)
N1—P1—O2—P1^i^	−61.51 (10)	C2—C3—C4—C5	−0.3 (5)
O3—P1—N1—C8	−28.6 (3)	C2—C3—C4—C7	179.7 (3)
O1—P1—N1—C8	−159.7 (2)	C3—C4—C5—C6	0.5 (5)
O2—P1—N1—C8	99.2 (2)	C7—C4—C5—C6	−179.4 (3)
O3—P1—N1—C9	−178.3 (2)	C2—C1—C6—C5	0.7 (4)
O1—P1—N1—C9	50.6 (2)	O1—C1—C6—C5	−175.4 (3)
O2—P1—N1—C9	−50.4 (3)	C4—C5—C6—C1	−0.7 (5)

Symmetry codes: (i) −*x*, *y*, −*z*+1/2.
